# Exploring Vocational Evaluation Practices following Traumatic Brain Injury

**DOI:** 10.1155/2015/924027

**Published:** 2015-09-30

**Authors:** Christina Dillahunt-Aspillaga, Tammy Jorgensen Smith, Ardis Hanson, Sarah Ehlke, Mary Stergiou-Kita, Charlotte G. Dixon, Davina Quichocho

**Affiliations:** ^1^Department of Rehabilitation and Mental Health Counseling, College of Behavioral and Community Sciences, University of South Florida, 13301 Bruce B Downs Boulevard, MHC 1632, Tampa, FL 33612-3807, USA; ^2^College of Behavioral and Community Sciences, University of South Florida, 13301 Bruce B Downs Boulevard, MHC 1139, Tampa, FL 33612-3807, USA; ^3^American Legacy Foundation, 1724 Massachusetts Avenue NW, Washington, DC 20036, USA; ^4^Department of Occupational Science and Occupational Therapy, University of Toronto, 160-500 University Avenue, Toronto, ON, Canada M5G 1V7; ^5^C.G. Dixon & Associates Inc., 42 S. Ingram Street, Alexandria, VA 22304, USA

## Abstract

*Background*. Individuals with traumatic brain injury (TBI) face many challenges when attempting to return to work (RTW). Vocational evaluation (VE) is a systematic process that involves assessment and appraisal of an individual's current work-related characteristics and abilities. *Objective*. The aims of this study are to (1) examine demographic and employment characteristics of vocational rehabilitation providers (VRPs), (2) identify the specific evaluation methods that are used in the VE of individuals with TBI, and (3) examine the differences in assessment method practices based upon evaluator assessment preferences. *Methods*. This exploratory case study used a forty-six-item online survey which was distributed to VRPs. *Results*. One hundred and nine VRPs accessed the survey. Of these, 74 completed the survey. A majority of respondents were female (79.7%), Caucasian (71.6%), and holding a master's degree (74.3%), and more than half (56.8%) were employed as state vocational rehabilitation counselors (VRCs). In addition, over two-thirds (67.6%) were certified rehabilitation counselors (CRCs). Respondents reported using several specific tools and assessments during the VE process. *Conclusions*. Study findings reveal differences in use of and rationales for specific assessments amongst VRPs. Understanding VRP assessment practices and use of an evidence-based framework for VE following TBI may inform and improve VE practice.

## 1. Introduction

Traumatic brain injury (TBI) is a common injury with a unique incidence, prevalence, and consequence [1–4]. By definition, brain injury is “an insult to the skull, brain, or its covering, resulting from external trauma, which produces an altered state of consciousness or anatomic, motor, sensory, cognitive, or behavioral deficits” [5]. Individuals reporting any level of TBI severity, whether mild, moderate, or severe, have significantly higher percentages of activity limitations and lower satisfaction with life [6]. To determine the severity of TBI requires an assessment of patient function and observable structural properties of the affected brain [4, 7–9]. Some 3 to 5 million individuals in the United States currently live with the long-term effects of a TBI [4, 10, 11]. In Florida, where this study is located, over 210,000 people have a TBI-related disability and these numbers are expected to rise [12, 13].

TBI may affect any or all aspects of daily living, including the ability to work [2, 4, 14–26]. The national estimates of the costs of medical care, rehabilitation, and loss of productivity for persons with TBI approximate $76.5 billion annually [27–29]. Unemployment is higher among individuals with TBI, who often have significant problems working after injury [15, 30–42]. Approximately 60% of patients with TBI are unable to return to work and approximately 35% of individuals with TBI are able to find only part-time work (35%) [43]. Due to the consequential nature of the injury, returning to work for individuals with TBI is challenging [15, 17, 19–21, 27, 30–34, 40, 44–50]. Even individuals with mild traumatic brain injuries may experience limitations in employment and social functioning [39, 51].

Rates of unemployment are even higher (60–90%) for individuals with TBI who do not receive specialized rehabilitation or interventions [21]. In Florida, employment rates for individuals with TBI receiving state vocational rehabilitation services range between 8.6% and 10% [53, 54]. Underemployment and unemployment following TBI can have detrimental effects for individuals, their support systems, and their communities [55–57]. These include diminished life satisfaction and psychological well-being, as well as poor community reintegration in the areas of home, social, and leisure activities [2, 36, 58–60].

Conversely, there are many benefits in returning to work following a TBI. Individuals report improved quality of life and fewer symptoms of depression and anxiety [26, 34, 50, 58, 61]. Work also provides a sense of structure and purpose, has economic rewards, and helps maintain respect from peers [32, 49]. Essentially, work provides a sense of normalcy, allowing the individual to feel socially involved and connected after sustaining an injury [32, 49]. Thus, determining effective evaluations to help the person return to work is an important part of the treatment and rehabilitation of individuals with TBI.

Rehabilitation counselors and vocational rehabilitation professionals use a number of counseling and rehabilitative approaches to help persons with TBI make positive psychological adjustments to life in the community [16, 30, 32, 59, 62, 63]. Rigorous, comprehensive, and consistent vocational assessment and evaluation practices are essential for facilitating successful return to work for individuals with TBI [32, 59, 64]. Vocational assessments identify an individual's characteristics, education, training, and placement needs; serve as the basis for planning an individual's educational program; and provide insight into his or her vocational potential [65, 66].

There are three levels within a vocational assessment [65, 67, 68]. Level one screens for additional services and captures necessary, relevant, and appropriate information. Level two is the clinical phase and involves detailed case study, interviews, in-depth vocational counseling, and psychometric testing. It also may include a transferrable skills analysis. Level three is the final and most comprehensive level, which includes the vocational evaluation process. Level three is an extension of level two and may include additional strategies, such as job analysis, work samples, situational and community based-assessments, and observation of real and simulated work behavior [65, 67, 68].

Specifically, vocational evaluation is defined as a comprehensive, collaborative, interprofessional process of evaluating an individual's current work abilities and work functions, limitations, and tolerances in order to (a) gain an understanding of an individual's work-related strengths and deficits, (b) determine whether the occupation or job being evaluated is consistent with the individual's interests and abilities, and (c) make recommendations as to the supports necessary to achieve the identified occupational or job goal (e.g., training, education, job coaching, additional services, and supports) [65, 68]. A review of the literature reveals that no evaluative or randomized controlled trials examining the effectiveness of specific vocational assessment or evaluation practices following TBI currently exist [17, 32]. However, empirically validated neuropsychologically based vocational batteries, such as the McCarron-Dial System (MDS) [69–72], do exist.

**Figure 1:**
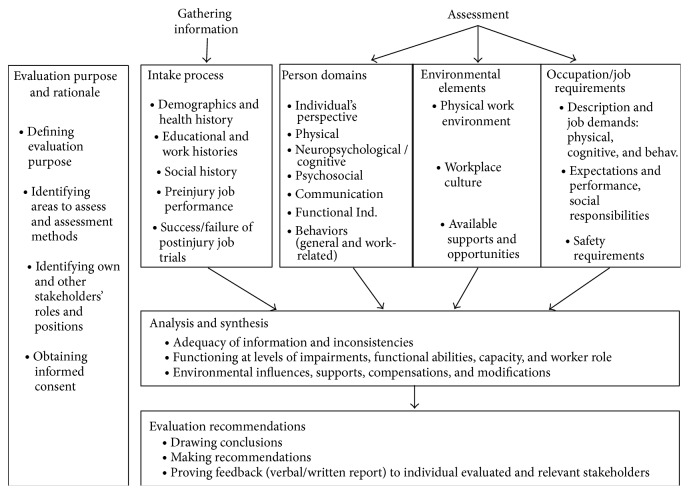
Evidence-based framework for vocational evaluation following TBI. This figure was reprinted with kind permission from Springer Science and Business Media. Note: reprinted with permission CCO license number 3697230252812. The figure was originally published in [52].

Until recently, there were no specific detailed guidelines for VE of cases involving TBI [31, 32, 64, 67]. However, Stergiou-Kita and colleagues [64] identify seven process domains, with key factors integrated in each domain, evaluators should utilize when they conduct VE with individuals with TBI. The process domains are (1) identification of the evaluation purpose and rationale; (2) intake process; (3) assessment of person; (4) assessment of the environment; (5) assessment of occupation/job requirements; (6) analysis and synthesis of assessment results; and (7) development of evaluation recommendations. Key personal, environmental, and occupational factors also are considered within the context of their effects on an individual's work performance. These domain processes and factors are integrated into an evidence-based framework (EBF), which was utilized to develop the clinical practice guideline (CPG) for VE following TBI. The purpose of the EBF following TBI is to outline what important information vocational evaluators should consider, discuss, and recommend during and after completion of VE.  Figure 1 illustrates the EBF for VE following TBI [52, 64].

Considering the increase in TBI nationally and in Florida, and the development of a VE clinical practice guideline specific to TBI, there is little in the literature that examines the daily practice of VE for TBI. With that in mind, we conducted a case study to gain a fuller understanding of how vocational rehabilitation professionals make sense of the VE process when evaluating a person with TBI. Hence, the purpose of this paper is threefold. First, the study describes demographic and employment characteristics of a small cohort of vocational rehabilitation providers (VRPs) who evaluate individual work abilities following TBI. Second, the study broadly identifies the evaluation methods reported as important when conducting vocational evaluations with individuals with TBI after injury. Finally, it examines differences in preferred tools and assessments used by evaluators.

## 2. Methods

This case study explores the processes and factors relevant to the practice of VE following TBI in the state of Florida with the intent to (1) inform current vocational evaluation practices and (2) improve the understanding of vocational assessment and evaluation of individuals with TBI.

### 2.1. Ethics Statement

The Institutional Review Board (IRB) determined that this research study (eIRB 00013147) met the University of South Florida (USF) requirements and Federal Exemption criteria as outlined in 45 CFR §46.101(b)(2): “research involving the use of educational tests (cognitive, diagnostic, aptitude, achievement), survey procedures, interview procedures or observation of public behavior; unless (i) information obtained is recorded in such a manner that human subjects can be identified, directly or through identifiers linked to the subjects; and (ii) any disclosure of the human subjects' responses outside the research could reasonably place the subjects at risk of criminal or civil liability or be damaging to the subjects' financial standing, employability, or reputation.” Before responding to any study questions, participants completed an electronic online informed consent form.

### 2.2. Design

Case study designs focus on providing a detailed account of one or more cases with an interest in both their uniqueness and commonality. Since the intent of a case study is to be descriptive, exploratory, and explanatory, case studies are preferred when we want to make sense of a situation or how an individual understands something that is not readily apparent to an external viewer. Understanding the “how” or “why” things are done addresses a “phenomenon” within a real-life context and can be used as a sense-making tool that can help change practice and inform policy. Case study designs are often used when conducting exploratory or pilot studies of a particular population.

The target population was vocational rehabilitation providers (VRPs) in Florida who conduct and/or review vocational evaluations of individuals with TBI who reside in Florida. The VRP included public and private rehabilitation counselors and independent vocational evaluators and vendors in Florida. This one-year exploratory study distributed a cross-sectional online survey to VRP. A web-based survey format was chosen to provide a “snapshot” of the target population and to establish a baseline of their perceptions [73]. The survey was anonymous and self-administered online and contained forty-six (46) items. Survey questions were a mix of yes/no, single and/or multiple answer selections, Likert scales, and open-ended questions. Many of the yes/no questions included an option for further comments or explanations.

Survey items were developed based on an empirically validated framework for vocational evaluation following TBI [52, 64]. Survey items captured basic demographic information about VRP, including age, sex, race/ethnicity, level of education, and history of employment. Items further surveyed evaluation processes perceived to be most valuable and identified the tools evaluators used to conduct or review assessments. Continuous and categorical scales were used to measure items on the survey instrument. Stratification was not used before selecting the sample.

The anonymous survey function in the Qualtrics Survey Research Suite ensured that all responses were anonymous. Further, since Qualtrics uses cookies to save the respondents' progress, if the respondents start the survey, leave, and come back to the same browser, they will resume where they left off [74]. Settings in Qualtrics that were selected did not allow respondents to go back and change answers, but they were allowed to skip questions. Participants could complete the survey from their computer. The survey could be accessed from other computers and locations.

The initial survey was sent to two experts in vocational evaluation and survey design for review, to solicit feedback and to provide an estimate of survey completion time. Minor suggestions for reorganization and editing were provided by the experts. As a result, minor modifications and improvements were made prior to distributing the survey to VRPs in Florida. Due to the length of the survey, it is not included in the appendix. The corresponding author will provide a copy of the survey upon email request.

### 2.3. Participants

Participants in the current study were Florida VRPs who were invited (via email) to participate in an online survey to assess factors related to vocational evaluations (VEs) of individuals with TBI. The invitational email included a description of the study and provided a link to an electronic online informed consent form. The electronic consent form, approved by the USF IRB, ensured that participants would have appropriate information on the scope and aims of the study, as well as the procedures the researchers would use to ensure confidentiality and privacy of the respondents.

Email addresses were obtained from the Florida Division of Vocational Rehabilitation (FL-DVR), the Commission on Certification of Rehabilitation Counselors (CRCC), and the International Association of Rehabilitation Professionals (IARP). To be included in the study, VRP must currently conduct or review vocational evaluations of individuals with TBI residing in the state of Florida or must have conducted or reviewed vocational VEs within the past five years.

VRP respondents were excluded from the study if they (1) had their certification or licensure revoked; (2) were not currently conducting or reviewing VEs of individuals with TBI in Florida; (3) had not conducted or reviewed VEs with individuals within the previous five years; (4) had medical or psychiatric conditions precluding comprehension/completion of the study; (5) were students without appropriate certification and licensure; or (6) were unable to provide informed consent.

Preapproval to distribute the survey to vocational rehabilitation professionals was obtained from the Director of the Division of Vocational Rehabilitation in Florida. A total of 653 emails were sent to public and private VRPs in Florida. Of the possible number of total respondents, 109 respondents accessed the survey and 80 (73.3%) respondents completed the online survey. Six individuals did not currently work with individuals with TBI or they had not worked with this population within the past five years. Since these 6 individuals were not eligible to complete the study, no demographic information was collected on them. The final sample used for the present study was 74 respondents. There were no differences in sociodemographic variables between those who did (*N* = 74) and did not complete the survey (*N* = 29). All respondents (including those who did not meet the inclusion criteria) were linked to a separate survey to request a copy of the survey report and enter the drawing for a chance to win the gift card.

There are many potential influences on response rates in e-mail surveys, including respondent contacts, length of surveys, design issues, research and academic affiliations, and compensation [75]. Kaplowitz et al., for example, determined that there was a 10 percentage point discrepancy in the number of responses to an email survey (21%), compared to a postal survey (31%) [76]. Since our intent was to confirm that the sampling process acquired a representative collection of respondents for purposes of a case study and as a pilot study, the 17% response rate was low but deemed acceptable. The average survey completion time was 20 minutes, which was five minutes longer than the time estimated by both of our expert survey reviewers. This discrepancy may be due to the experts' familiarity with the subject matter and the Qualtrics survey research program.

### 2.4. Procedure

Participants who responded to the invitational email completed a 15-minute online survey administered through the Qualtrics software program, which administers surveys and stores confidential survey responses. Qualtrics data are stored in SSAE 16 certified facilities that meet the privacy standards imposed on health care records by the Health Insurance Portability and Accountability Act and the Health Information Technology for Economic and Clinical Health Act [77].

The online survey consisted of 46 yes/no, multiple choice, and open-ended questions. In addition to the collection of basic demographic information, items asked about specific evaluation processes, tools used when conducting VEs, and the review of client vocational reports. Survey items were developed based upon the existing clinical practice guideline entitled* Evidence-Based Framework for Vocational Evaluation following TBI (EBF)*. Survey items were aligned with the seven processes in the* EBF* [52, 64]: (1) identification of the evaluation purpose and rationale; (2) intake (gathering information); (3) person domains (assessment); (4) environmental elements (assessment); (5) occupation and job requirements (assessment); (6) analysis and synthesis; and (7) evaluation recommendations.  Figure 1 illustrates the evidence-based framework for VE following TBI [52, 64].

As a recruitment strategy, participants had the option to sign up for a chance to win a $100 gift card after they completed the survey. If they were interested in entering the drawing, respondents were redirected to a separate survey to provide their contact information. To ensure confidentiality, the separate survey was not linked to the main survey.

### 2.5. Data Analysis

All data responses were downloaded from the Qualtrics website into an SPSS 22.0 dataset file. Data included demographic responses, tools and assessments used in VE, specific characteristics of VRP, and responses to the open-ended questions. Demographic responses were coded and grouped based on the level of education, certification, and training of the respondent. Respondents reported their highest level of education (bachelor's, master's, or doctorate degree) and what type of certifications they currently hold (respondents were able to select more than one certification or credential). In addition, respondents reported how many years of experience they had conducting VE by selecting the appropriate range of years of experience in the survey.

Qualitative data were gathered through open-ended survey questions. The questions were used to (1) clarify the choice of predefined responses and (2) gather additional qualitative data on the purpose of the VE. The questions included the purpose and rationale of the VE; the intake process; issues surrounding assessment, data analysis, and synthesis; and evaluation recommendations. Respondents also were asked to consider characteristics of best TBI evaluators and list characteristics that set them apart from other TBI evaluators. The qualitative comments were coded, and responses were grouped into thematic categories identified in the* EBF*: evaluation purpose and rationale, gathering information, assessment, analysis and synthesis, and evaluation recommendations.

One question in particular, characteristics of the best TBI evaluators, was subjected to a more granular analysis based upon researcher consensus that these characteristics may be indicative of model VRP practice. This question was subjected to a second thematic analysis of eight categories: use of detailed reports, knowledge/experience with TBI population, client interaction, individualized assessment, job search information, quick evaluations, understanding the purpose of the evaluation, and creativity/honesty.

## 3. Results

### 3.1. Descriptive Information

#### 3.1.1. Demographic Data

The majority of the participants were female (79.7), white (71.6%), and holding a master's degree (74.3%). Additionally, the majority were certified rehabilitation counselors (CRCs) (67.6%); over half (56.8%) were employed as state vocational rehabilitation counselors (VRCs). Tables 1 and 2 display the demographic data.

#### 3.1.2. Tools, Assessments, and Techniques Considered and Used When Conducting VE

Table 3 shows the variety of tools, assessments, and techniques used when conducting VE. The most prevalent tools used were vocational interest inventories (86.5%), achievement tests (85.1%), and behavioral observations (81.1%). All other prevalence estimates are reported in Table 3.

#### 3.1.3. Important Characteristics of TBI Vocational Evaluators

Respondents qualitatively described characteristics of best TBI evaluators and listed characteristics that set them apart from other TBI evaluators. Participant responses were grouped into eight categories. Over a quarter (27.0%) of the respondents believed that having knowledge of TBI and having experience working with this population were important (27.0%); 24.3% believed that including detailed evaluation reports of the client in the evaluation was important. Additionally, 21.6% believed it was equally important to have positive interactions with the client and also to provide individualized assessments. A smaller proportion of respondents believed that providing the client with job search information (5.4%), being creative and honest when considering job prospects (5.4%), and providing abbreviated (4–6 hours versus 8 hours) evaluations (4.1%) were important characteristics of a VRP. However, understanding the purpose of the evaluation (1.4%) was noted as the least important characteristic to consider during the vocational evaluation of individuals with TBI (Table 4).

## 4. Discussion

In this study, an evidence-based framework for VE following TBI guided the development of a survey that explored the current practice of the vocational evaluation of individuals with TBI in Florida. Consistent with previous research, the data in this study suggest that VRPs vary in their preference for and use of tools, assessments, and techniques during the vocational evaluation process [32, 52, 64, 78]. Survey data further elucidates how these variations can affect VE practice.

### 4.1. General Survey Feedback

In this section, we examine the open-ended comments provided by the respondents, using selected elements of the framework from the* Evidence-Based Framework for Vocational Evaluation following TBI*, as shown in Figure 1: evaluation purpose and rationale, gathering information, assessment, analysis and synthesis, and evaluation recommendations [52]. Incorporated into this analysis are data describing important characteristics of TBI vocational evaluators.

### 4.2. Evaluation Purpose and Rationale

#### 4.2.1. Defining Evaluation Purpose

Stergiou-Kita and colleagues [52, 64] suggest that an essential domain for VE was the identification of the evaluation purpose and rationale. Although only four respondents (1.4%) explicitly stated this was an important characteristic for persons performing VE, the open-ended comments suggest otherwise. Respondents provided a wide range of definitions, such as “the purpose of a vocational evaluation is to learn about the customer's interests, strengths, and aptitudes.” We also get an idea of the person's functional limitations and strengths. In this way we are able to develop a mutually agreeable vocational goal based upon a person's unique needs and strengths. They also delved into elements that may play a factor in VE, including identification of discrete items, such as hard and soft work skills; additional accommodations, services, and supports; and “transferable” skills, abilities, and/or interests. These items, which come from the evaluation, assist in determining “achievable and appropriate employment” for the client, which is central to VE.

### 4.3. Identifying Own and Other Stakeholder's Roles and Positions

Our finding that VRPs (27.0%) value the importance of experience and knowledge working when evaluating persons with TBI mirrors findings in the literature [30, 52, 79]. Naturally, experience may benefit vocational evaluators in all areas. However, experience and knowledge particularly contribute to a VRP's understanding of the complexity of TBI in helping their clients return to work. One respondent offered, “My clinical background and knowledge regarding the medical aspects of disability give me a unique perspective regarding the vocational impact of TBI.” Additionally, it is important for evaluators to be familiar with challenges and obstacles that clients may face following a TBI. Another respondent stated that knowledge of individuals with disabilities helped clients identify their positive attributes and strengths, which tend to be harder for clients to identify than weaknesses. By being knowledgeable in these areas, VRP may be able to better support client/community reintegration and affect the likelihood of employment [80].

### 4.4. Identifying Areas to Assess and Assessment Methods

Respondents expressed the importance of providing detailed reports. The more detailed vocational evaluation reports are, the better they may assist counselors in determining the type and intensity of vocational services needed for their clients [68]. Respondents further highlighted the importance of evaluators having positive interactions with clients (21.6%) and conducting individualized assessments (21.6%). By providing individualized assessments, individuals with TBI may feel that they have established rapport with evaluators who are genuinely vested in their interests. Respondents agreed that “a client's personal strengths, preferences, and family considerations” were central in individualized assessments. Respondents were less likely to view abbreviated evaluations as helpful. “Tailored,” “appropriate,” “practical,” and “realistic” were terms consistently used to describe both vocational and other client's evaluations. This may be due to a preference to spend more time discussing the purpose of the evaluation with the client and to gather detailed background information (e.g., health, social, and work histories) during the evaluation to better meet client- and VRP-generated goals [52, 57, 59, 80].

### 4.5. Assessment (Person, Environment, and Occupation/Job)

In the literature, rehabilitation counselors consider the assessment of physical, cognitive, and psychosocial abilities, work interests, and work behaviors to be extremely important [62, 66, 68]. Almost a quarter of our respondents concurred (24.3%). Respondents indicated that they most frequently assess individuals through behavioral observation, interest inventories, work values inventories, and achievement tests. As one respondent noted, VE should “have and use a wide variety of instruments” and be able “to explain WHY they used the testing instruments that they did.” However, respondents placed less emphasis on assessing communication skills, workplace culture, and social support networks. This is problematic as individuals' communication skills have been found to be relevant to employment outcomes following TBI [81]. However, respondents did identify assistive technologies and compensatory strategies as important components for assessment and augmentation of existing deficits, especially as supports from a workplace perspective. Understanding workplace culture elements and identifying the supports that are available, to individuals with TBI, both within the workplace and their support networks, can provide the rehabilitation counselor with further valuable information for ensuring successful work transitions [15, 33, 48, 64, 80].

Respondents also reported several types of assessments to be particularly beneficial during conducting and reviewing vocational evaluations following TBI. These included situational assessments, community assessments, cognitive demands analyses, and job shadowing, which parallels findings in other studies [30, 64, 66, 79, 82, 83]. However, we also found that a very small percentage (5.4%) of VRPs use the McCarron-Dial System (MDS). Although only four individuals reported using the MDS, this finding is interesting because research shows the MDS is effective as a vocational evaluation system for persons with neuropsychological disabilities, such as TBI [72, 84]. There are several factors that may contribute to low use of this reliable and valid assessment. One factor may be the lack of knowledge about the MDS by vocational evaluators. Additional factors may be related to the cost of the system, training requirements (three dedicated days), and the amount of time needed to administer the full battery (1 week) or the abbreviated version (one half day). Further research on the potential usefulness of incorporation of the MDS into vocational rehabilitation counselor (VRC) training and direct service settings may be timely, warranted, and beneficial [85].

Slightly over half (52.7%) of study participants refer to neuropsychological reports. The literature suggests that vocational rehabilitation counselors (VRCs) may have difficulty in understanding how to use and interpret neuropsychological tests or how they apply to vocational preparation and return to work [44, 81, 86–88]. The literature, however, also shows an increase in the number of vocational rehabilitation counselors relying on neuropsychological reports to better assess their clients for job placement [64, 79, 86]. Use of neuropsychological reposts by VRP is an area of research that is not yet fully elucidated and deserves further study. Neuropsychological reports may reveal additional information that the counselor is unable to capture during qualitative interviews with the client or through review of traditional vocational evaluation reports [70, 88]. Therefore, a combination of assessment techniques and a synthesis of findings from a variety of reports may be beneficial when assisting individuals with TBI in their return to work.

### 4.6. Analysis and Synthesis

Overall, respondents concurred that analysis and synthesis of results are two important pieces of VE; however, they are also very difficult. As mentioned above, being able to explain why specific testing instruments were used and how the results are interpreted to the client when making workplace recommendations is essential, but difficult. Reports need to be “thorough and individualized” and analyses need to factor the information necessary to address client concerns. One respondent suggested “bluntness with professional tact in conveying the realities of TBI disabilities upon placement” is a necessary and valuable characteristic for VRP, when analyzing and synthesizing all of the measures and assessments used in VE.

When asked about additional assessments for VE, respondents provided a number of areas they would like to have. Pre- and postinjury functioning were mentioned, as that would provide a 360° perspective on the client's work experience and expectation for VE. “Reality check” questions, health care needs questions, support system questions, and motivation questions were also pointed out as essential for assessment. One recurring theme was the “ability to use all aspects of the evaluation to develop the report.”

### 4.7. Evaluation Recommendations

According to respondents, recommendations should not be just the “examination of abilities and aptitudes, but also include the true feedback of the client.” Respondents also were clear that information should be presented in an easy to read manner and, perhaps, most importantly, recommendations should be a working document that “counselor and consumer can agree on and work towards.”

Some findings deviated from previous literature in the field. Unlike previous findings which suggest that providing prospect and career advice is an important component of VE [52, 80, 89], only 5.4% of our respondents explicitly emphasized the importance of providing clients with honest, direct, and creative information for job prospects and information on how to improve the job search. However, respondents did identify truthfulness, honest, and accurate assessment, evidence-based vocational recommendations, and knowledge of the local labor market as important characteristics for vocational evaluators. Also, respondents may view the VE as the initial step in the return-to-work process and that the information gathered during the evaluation would contribute to future suggestions and strategies to support successful job searches. Respondents also may believe that they already provide this type of information as part of their daily practice and saw no reason to emphasize this in their responses. In retrospect, the survey should have explicitly asked this question. However, we extrapolate, based on the open-ended responses, that client needs and the information to help clients succeed are very important.

### 4.8. Limitations

Considerations should be taken when interpreting the results of this study. Limitations of the study include a relatively small sample size, including mostly VRP who work for the state of Florida Division of Vocational Rehabilitation. A larger and more representative sample is needed to contribute to the validity, reliability, and significance associated with these findings. Since the sample consisted predominantly of Caucasian females who hold master's degrees and are CRCs, this sample did not represent diversity in culture and gender of individuals who work as evaluators in the field of vocational rehabilitation. An additional limitation is the survey instrument, which lacks baseline measures.

Future research should include refinement of the survey instrument and consider incorporating more formal measures to examine additional factors, such as alcohol and substance use, cooccurring disorders, use of medications, and personality assessments, and consider salient factors affecting job satisfaction and job tenure. The Qualtrics survey settings allowed for survey access and completion from different computers. When offering an incentive it is important to prevent participants from taking a survey more than once. Although there is no evidence that this occurred in our study, Qualtrics' “Prevent Ballot Box Stuffing” option will prevent respondents from taking future surveys more than once from the same computer. Future use of the Qualtrics Mailer (which creates a unique, one-time use link for each participant) will also prevent respondents from completing the survey more than once. Finally, the study was sent once via email to VRP in the state of Florida. Future recruitment efforts should include mechanisms to expand access to a more representative sample that is generalizable to a broader population of VRP who work with individuals with TBI outside of Florida.

## 5. Conclusions

In summary, return to work for individuals with TBI is complex and challenging due to the consequential nature of the injury. Despite the provision of necessary rehabilitation supports, many of these individuals encounter a myriad of barriers that impede successful vocational rehabilitation [62]. Employment rates are lower for persons with TBI compared to those of the general population [43, 68, 77]. Considering the complexity of challenges individuals with TBI face, rehabilitation counselors, and other vocational rehabilitation providers should consider expanding their understanding of TBI and incorporate the use of specific skills, techniques, and tools to provide psychosocial and vocational supports to facilitate return to work [62]. Due to the variability in vocational evaluation of individuals with TBI, vocational rehabilitation counselors (VRCs) and providers need to understand the key processes and relevant factors important for a thorough and rigorous vocational evaluation. VRPs require a clear, clinical knowledge of the skills and abilities that are being measured, technical expertise in test/assessment procedures, and good understanding of and appreciation of the demands of the position and work environment the client may be entering. The introduction, adoption, and successful implementation of new technologies and assessments in vocational evaluation and rehabilitation should be a focal content area in continuing education and professional development activities as well as statewide policy initiatives taking research to practice.

The evidence-based framework for VE following TBI presented may be useful for rehabilitation educators, counselors, vocational evaluators, and other rehabilitation providers. These findings can inform the current practice of vocational evaluation and ultimately improve return-to-work outcomes for individuals with TBI, by guiding and improving the vocational assessment process. Further research is needed to formally examine the success of the framework among a diverse group of vocational rehabilitation providers.

## Figures and Tables

**Table 1:** Demographic information (*N* = 74).

Variable	*N* (%)
Gender	
Female	59 (79.7)
Male	14 (18.9)
Did not answer	1 (1.4)
Age group	
18–30	13 (17.6)
31–40	19 (25.7)
41–50	14 (18.9)
51–60	16 (21.6)
>61	9 (12.1)
Did not provide a response	3 (4.1)
Race	
White	53 (71.6)
Black/African American	9 (12.2)
Hispanic	7 (9.5)
Other^*∗*^	5 (6.7)
Highest education	
Bachelor's degree	13 (17.6)
Master's degree	55 (74.3)
Doctoral degree	6 (8.1)

*Note*. ^*∗*^Other includes respondents that were either biracial (2), Asian/Pacific Islander (1), or Native American (2).

**Table 2:** Employment information: credentials, years of experience, and occupation (*N* = 74).

Variable	*N* (%)
Credentials^*∗*^	
CAP	3 (4.1)
CCM	4 (5.4)
CDMS	1 (1.4)
CLCP	2 (2.7)
CRC	50 (67.6)
CVE	14 (18.9)
LMFT	2 (2.7)
LMHC	7 (9.5)
ABVE	3 (4.1)
PVE	6 (8.1)
Other^*∗∗*^	9 (12.2)
Years of vocational rehabilitation experience	
<1 years	3 (4.1)
1–5 years	16 (21.6)
5–10 years	23 (31.1)
10+ years	32 (43.2)
Occupation^*∗*^	
State VR counselor	42 (56.8)
Rehabilitation educator	3 (4.1)
Independent rehabilitation Provider, public sector	3 (4.1)
Independent rehabilitation Provider, private sector	11 (14.9)
Rehabilitation service provider, nonprofit	6 (8.1)
Rehabilitation service provider, for profit	3 (4.1)
Other^*∗∗∗*^	21 (28.4)

*Note*. ^*∗*^Respondents may choose more than one category. ^*∗∗*^Other examples include Florida Certified Workforce Professional, Mental Health Counseling Intern, National Certified Counselor, Certified Brain Injury Specialist, Certified Work Adjustment Specialist, Certified Psychiatric Rehabilitation Practitioner, Certified Multisystemic Therapist, and Certified Clinical Mental Health Counselor. ^*∗∗∗*^Other includes VR area supervisor, VR field supervisor, Division of Blind Services supervisor, veteran rehabilitation counselor, consultant, forensic vocational expert, and vocational evaluator not otherwise specified.

**Table 3:** Tools/assessments/techniques used in vocational evaluation.

Variable^**∗**^	*N* (%)
Learning style preferences	30 (40.5)
Vocational interest inventories	64 (86.5)
Work values	53 (71.6)
Evaluator knowledge of TBI	54 (73.0)
Behavioral observations	60 (81.1)
McCarron-Dial System	4 (5.4)
Achievement tests	63 (85.1)
Aptitude tests, general abilities	45 (60.8)
Work samples	46 (62.2)
Neuropsychological evaluation reports	39 (52.7)
Aptitude tests, intelligence	52 (70.3)
Computer software/training aids^*∗∗*^	15 (20.3)
Other^*∗∗∗*^	13 (17.6)

*Note*. ^*∗*^Respondents may choose more than one tool/assessment/technique. ^*∗∗*^Brain Train was specifically mentioned by respondents. ^*∗∗∗*^Other = situational assessment, job site analysis, customized employment (e.g., Discovery), on-the-job training, volunteering, personality assessments, input from past employers and family members, and Veteran TBI Clinical Assessment.

**Table 4:** Important characteristics of successful TBI vocational evaluators.

Variable^*∗*^	*N* (%)
(1) Detailed reports	18 (24.3)
(2) Knowledge of TBI/experience	20 (27.0)
(3) Interaction with client	16 (21.6)
(4) Individualized assessment	16 (21.6)
(5) Job search	4 (5.4)
(6) Quick evaluations	3 (4.1)
(7) Understanding the purpose of the evaluation	1 (1.4)
(8) Creative/honest	4 (5.4)

*Note*. ^*∗*^(1) Providing detailed reports, (2) having knowledge and experience working with the TBI population, (3) having positive interactions with the client, (4) providing individualized assessments, (5) providing the client with job search information, (6) conducting quick yet thorough evaluations (4-5 hours in duration), (7) having a good understanding of the purpose of the evaluation, (8) and being honest, open-minded, and having creative ideas to help accommodate the client.
